# Intraoperative glycemic control using an artificial endocrine pancreas in a patient with a recurrent pleural solitary fibrous tumor producing insulin-like growth factor 2: a case report

**DOI:** 10.1186/s40981-019-0229-y

**Published:** 2019-02-05

**Authors:** Kenzaburou Sugimoto, Rina Tokitou, Mamoru Kadosaki, Mamoru Takeuchi

**Affiliations:** Department of Anesthesiology and Critical Care Medicine, Jichi Medical University, 3311-1, Yakushiji, Shimotsuke City, Tochigi 329-0498 Japan

**Keywords:** Solitary fibrous tumor, Artificial endocrine pancreas, Insulin-like growth factor 2, Non-islet cell tumor hypoglycemia

## Abstract

**Background:**

Non-islet cell tumor producing insulin-like growth factor 2 involves hypoglycemia. During tumor resection, intense fluctuation of blood glucose level may occur. An artificial endocrine pancreas has been reported as beneficial for patients with insulinoma as it maintains stable glycemic levels, although scarcely described with insulin-like growth factor 2-releasing tumor.

**Case presentation:**

An 84-year-old man had a recurrent left pleural solitary fibrous tumor releasing high molecular weight insulin-like growth factor 2 and experienced a frequent syncope accompanied by hypoglycemia. After anesthesia induction, an artificial endocrine pancreas, STG-55, was connected to the patient. Blood glucose level was stable at around 150 mg/dl during the resection surgery. The patient followed an uneventful course and was discharged without any complications.

**Conclusions:**

An artificial endocrine pancreas may have the potential to stabilize the intraoperative blood glucose change in insulin-like growth factor 2-releasing tumor resection.

## Background

Hypoglycemia mostly originates from diabetic therapy, including insulin and its secretagogues. Hypoglycemia can also occur due to inanition, hepatic disorders, hormone deficiencies, antibodies to insulin or its receptor, performance of gastric bypass, and tumors (i.e., tumor-induced hypoglycemia [TIH]) [1]. The most common cause of TIH is insulinoma, an insulin-hypersecreting tumor of pancreatic islet beta cells [2]. Non-islet cell tumors also secrete excess insulin or insulin-like hormones, resulting in non-islet cell tumor hypoglycemia (NICTH). The main etiology of NICTH is the release of insulin-like growth factor 2 (IGF2) [2]. Surgical resection is the most curative therapy for NICTH, although intraoperative glycemic control is quite challenging for anesthesiologists because of intense fluctuation of the blood glucose level. An artificial endocrine pancreas (AP) is capable of continuous measurement of the blood glucose level with automatic adjustment of the insulin or glucose infusion rate to achieve stable glycemic control. Its efficacy has been reported in patients with insulinoma [3], although its benefits in patients with NICTH with IGF2 elevation remain unclear. We herein report a case involving resection of an IGF2-producing pleural solitary fibrous tumor (SFT) using an AP.

## Case presentation

An 84-year-old man presented with frequent episodes of syncope due to hypoglycemia and was diagnosed as a recurrent left pleural SFT. He underwent the left pleural malignant SFT resection with uneventful course at the age of 75 years. The laboratory examination before the first surgery demonstrated a fasting plasma glucose level of 43 mg/dl. The low immunoreactive insulin (IRI) level of 9.5 μU/ml at 2 h after a meal indicated suppressed insulin secretion, and the western blot revealed an elevated plasma level of high molecular weight IGF2.

**Fig. 1:**
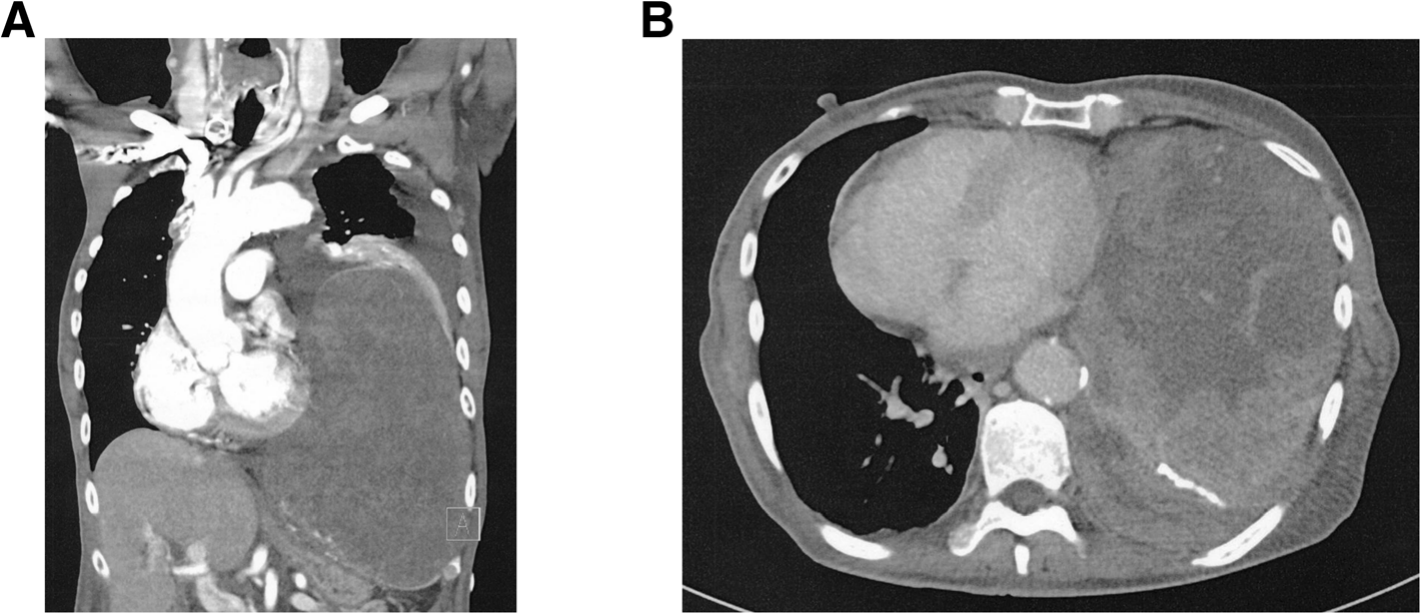
Preoperative computed tomography scan. **a** Coronal plane; a giant tumor occupied patient’s left pleural cavity and compressed the lung. **b** Axial plane

On admission for the second surgery, his height and weight were 169 cm and 66 kg, respectively. His medications included antagonists of aldosterone, calcium, and alpha-adrenergic receptor. His blood pressure and heart rate were 120/58 mmHg and 90 beats/min, respectively. He had a giant left pleural mass (11 × 16 × 22 cm; Fig. 1) and exhibited dyspnea. He had oral intake during the day and received continuous glucose infusion at a rate of 0.2 g/kg/h at night. His glucose level in the early morning sometimes decreased to approximately 30 mg/dl; thus, glucose boluses were administered as needed.

**Fig. 2:**
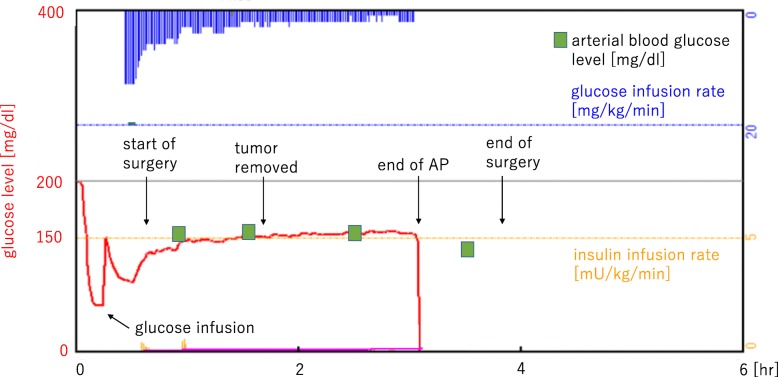
A glycemic change (red) and the alteration of glucose (blue) and insulin (yellow) infusion rate during the tumor resection. AP artificial endocrine pancreas

**Fig. 3:**
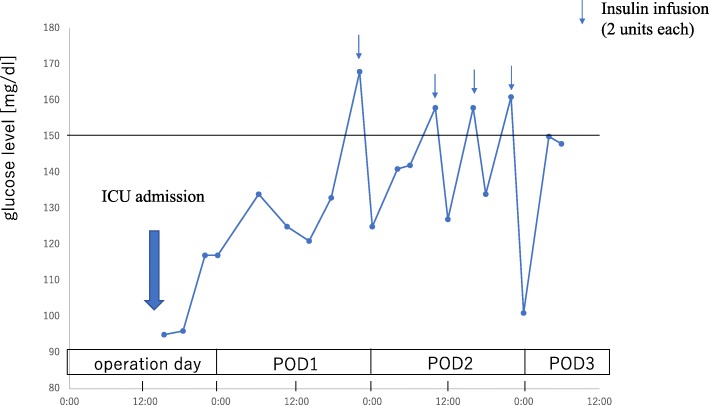
Postoperative glycemic change. Insulin infusion was administered in case the blood glucose levels were over 150 mg/dl. ICU intensive care unit, POD postoperative day

Anesthesia was induced with propofol, fentanyl, and rocuronium and maintained with propofol, remifentanil, and rocuronium. The radial artery and internal jugular vein were catheterized to monitor the arterial and central venous pressure, respectively. After induction, a 20-gauge peripheral catheter (Becton Dickinson Insyte™, UT, USA) was inserted into a lower-extremity vein and connected to an AP, STG-55 (Nikkiso, Tokyo, Japan); continuous blood glucose measurement was then started. The target blood glucose concentration was set at approximately 150 mg/dl. The patient’s blood glucose concentration was 58 mg/dl immediately after anesthetic induction; then, we infused 10 g of glucose. Insulin (1 unit/ml) and 10% glucose were automatically and continuously infused from the central venous catheter. The blood glucose level was charted over time to visualize the patient’s glycemic fluctuation during the operation (Fig. 2). The blood glucose level was stable at around 150 mg/dl during surgery, while the difference between the glucose values of the AP and those measured by arterial blood sampling was within approximately 10 mg/dl. The operation time was 178 min, anesthesia time was 278 min, bleeding volume was 1190 ml, and packed red blood cell transfusion volume was 560 ml. The AP was disconnected from the patient at the end of the surgery. The postoperative blood glucose levels were measured 20 times for 4 days at intensive care unit, and totally, 8 units of insulin were administered (Fig. 3). After discharge to the ward, his glucose levels were measured 3 times a day within the normal range. Although he spent almost 10 days to start oral glucose intake and approximately 3 weeks undergoing rehabilitation, his postoperative course was uneventful and he was discharged with no complications.

## Discussion

SFTs are the most common cause of NICTH [4]. The main pathophysiology of NICTH is the excessive release of IGF2 or the high molecular weight IGF2 [2], an incompletely processed precursor of IGF2. IGF2 binds not only to the IGF receptor but also to the insulin receptor [5], exerting insulin-like activity, and suppressing insulin secretion, which leads to low fasting and postprandial IRI levels in these patients [4].

Hypoglycemia is well recognized as a major cause of coma and, in extreme cases, irreversible brain necrosis [6]. In contrast, hyperglycemia leads to the development of postoperative infection [7]. From this point of view, it is imperative that anesthesiologists avoid hyperglycemia and hypoglycemia during tumor resection. However, maintenance of euglycemia throughout surgery is quite challenging; intermittent blood glucose measurement cannot guarantee avoidance of hyperglycemia and hypoglycemia due to the time lag between recognition of the blood glucose level and administration of glucose or insulin. In addition, SFTs sometimes progress to a size of approximately 10 to 20 cm [4], and these giant tumors may compress the lungs and heart, resulting in hypotension or atelectasis. Moreover, the hypervascularity of the tumor may lead to a large amount of bleeding that necessitates massive transfusion. In such cases, anesthesiologists might not be able to concentrate on repeated checking of the blood glucose level with frequent adjustment of the glucose or insulin infusion rate, which is a laborious and time-consuming task.

An AP fulfills the requirement of continuous blood glucose measurement and promptly and automatically responds to the need for glucose or insulin infusion without delay, thus achieving stable target glycemic values [8]. The device is reportedly beneficial for patients with insulinoma in that it rapidly responds to changes in the glucose level and obtains stable glycemic control [3]. The present case implicates that an AP is likely to be efficacious during the surgery even in patients with NICTH, although further case reports are needed to fully elucidate its effectiveness. The reliability and accuracy of the blood glucose values obtained from the AP have been described [9]. In our case, the difference between the values obtained from the AP and those obtained from arterial blood sampling was within 10 mg/dl, an acceptable range.

Regarding the preoperative glycemic management, our patient’s oral glucose intake during the daytime sufficiently avoided serious hypoglycemia because he could adjust the amount and the timing of food by himself; thus, oral glucose intake is recommended to avoid hypoglycemia before surgery [4]. In case patients are unable to take food by mouth due to compromised physical status or digestive disorders before surgery, the use of an AP may aid in achieving stable glycemic control and eventually may lead to the reduction of hypoglycemic brain damage and postoperative infection.

The blood glucose level occasionally tends to increase after insulinoma resection, a phenomenon known as rebound hyperglycemia [10] which is attributed to atrophy of the remaining pancreatic beta cells [11] or surgically induced pancreatic damage [10]. Our patient showed no signs of rebound hyperglycemia and required infrequent insulin boluses (Fig. 3). In patients with NICTH, the pancreas itself is intact and undamaged after surgery, and this fact may explain why no rebound hyperglycemia occurred in our patient.

In conclusion, our case suggests that an AP has the potential to stabilize the intraoperative blood glucose fluctuation that occurs in patients with NICTH involving IGF2-producing tumors.

## Abbreviations

AP: Artificial endocrine pancreas
IGF2: Insulin-like growth factor 2
IRI: Immunoreactive insulin
NICTH: Non-islet cell tumor hypoglycemia
SFT: Solitary fibrous tumor
TIH: Tumor-induced hypoglycemia
